# Sorption of Antibiotics in Sewage Sludge: Distribution Coefficients, Sludge Characteristics, and Implications for Environmental Fate

**DOI:** 10.3390/jox16030112

**Published:** 2026-06-14

**Authors:** Wonsik Shin, Pil-Gon Kim, Min-Ho Oak

**Affiliations:** 1 Division of Environmental Science and Ecological Engineering, Korea University, 145 Anam-ro, Seongbuk-gu, Seoul 02841, Republic of Korea; shinwg77@korea.ac.kr; 2 Convergence Center for Green Anti-Aging Research, Muan 58554, Republic of Korea; 3 Department of Environmental Education, Mokpo National University, Muan 58554, Republic of Korea; 4 College of Pharmacy, Mokpo National University, Muan 58554, Republic of Korea

**Keywords:** antibiotics, wastewater sludge, partition coefficient (*K_d_*), sorption, wastewater treatment plant

## Abstract

The sorption behavior of antibiotics in wastewater treatment systems plays a critical role in determining their environmental fate and removal efficiency. In this study, the sorption of 15 antibiotics representing multiple classes was investigated using two sewage sludge samples with different physicochemical characteristics. Batch equilibrium experiments were conducted to evaluate time-dependent sorption behavior and to determine solid–water distribution coefficients (*K_d_*). The results showed that sorption occurred rapidly, with most compounds approaching a stable concentration within 24 h. The *K_d_* values varied widely depending on the compound, ranging from 74 to 737 L/kg. For 13 of the 15 investigated antibiotics, higher *K_d_* values were observed in sludge B than in sludge A, with the largest difference observed for tiamulin (402 ± 53 and 737 ± 76 L/kg for sludge A and sludge B, respectively). Sludge B generally exhibited higher sorption capacity for most compounds than sludge A, despite having a lower specific surface area, indicating that sorption was governed primarily by chemical composition and pore structure rather than surface area alone. Elemental and morphological analyses suggested that differences in metal-associated components and pore structure may contribute to the higher sorption capacity observed in sludge B. However, the specific sorption mechanisms could not be directly confirmed by the present analyses. Comparison with previous studies confirmed that the measured *K_d_* values fall within reported ranges but are generally higher for sulfonamides, suggesting enhanced sorption capacity of the investigated sludge matrices. Application of an equilibrium-based model demonstrated that sorption alone can account for approximately 20–70% of antibiotic removal under typical activated sludge conditions, depending on compound affinity. These findings highlight the importance of sludge-specific properties in controlling antibiotic partitioning and demonstrate that incorporating such characteristics into predictive models can improve the accuracy of environmental fate assessments in wastewater treatment systems.

## 1. Introduction

The extensive use of antibiotics in human medicine and livestock production has led to their continuous release into the environment. A substantial fraction of administered antibiotics is excreted either unchanged or as biologically active metabolites and subsequently transported to wastewater treatment plants (WWTPs) via sewage systems [1,2]. Because conventional treatment processes are not specifically designed to remove such micropollutants, antibiotics have been widely detected in wastewater effluents, surface waters, and sewage sludge [3,4]. Sulfonamides, β-lactams, macrolides, tetracyclines, and fluoroquinolones are among the most frequently detected antibiotic classes in municipal wastewater and are considered environmentally relevant due to their persistence, ecological impacts, and potential contribution to the development and dissemination of antimicrobial resistance (AMR) [2,4,5,6,7,8,9,10,11]. Concentrations of antibiotics reported in municipal wastewater typically range from several ng/L to μg/L levels depending on consumption patterns, physicochemical properties, and treatment efficiency [1,2,4,5].

Within wastewater treatment plants, the fate of antibiotics is governed by a combination of biodegradation, transformation, and sorption processes. In addition, wastewater treatment plants are increasingly recognized as important reservoirs and dissemination pathways for antimicrobial resistance genes and resistant microorganisms, further increasing concerns regarding the environmental fate of antibiotics and their transformation products [4,6,7,8]. Various treatment technologies, including biological degradation, sorption onto activated sludge, membrane filtration, activated carbon adsorption, advanced oxidation processes, and electrocoagulation technologies, have been investigated for antibiotic removal from wastewater [4,5,9]. Nevertheless, sorption remains one of the dominant mechanisms governing the partitioning behavior of many antibiotics in conventional activated sludge systems.

The partitioning behavior of antibiotics is commonly described using the solid–water distribution coefficient (*K_d_*), which is a key parameter for predicting contaminant mobility and environmental fate [11,12]. However, the sorption behavior of antibiotics is more complex than that of conventional hydrophobic organic compounds, as it is influenced not only by hydrophobic interactions but also by electrostatic interactions, ion exchange, and complexation processes [13,14]. In general, strongly interacting compounds such as tetracyclines exhibit high sorption affinity, whereas sulfonamides tend to remain more mobile due to their relatively low sorption capacity [15]. In addition to compound-specific properties, the characteristics of sludge play a critical role in controlling sorption behavior. Sludge is a complex and heterogeneous matrix composed of organic matter, minerals, and microbial biomass, and its physicochemical properties can significantly influence contaminant partitioning [16]. Factors such as elemental composition, pore structure, and the presence of metal-associated components can affect sorption mechanisms and retention capacity. However, despite the recognized importance of these properties, the relationship between sludge characteristics and antibiotic sorption behavior has not been fully elucidated. Although numerous studies have investigated the occurrence and removal of antibiotics in wastewater treatment systems, comparatively few studies have experimentally determined *K_d_* values for multiple antibiotics using sewage sludge matrices with contrasting physicochemical characteristics. Furthermore, the influence of sludge pore structure and elemental composition on antibiotic sorption behavior has not been systematically evaluated. We hypothesized that differences in sludge physicochemical properties, particularly pore structure and elemental composition, would significantly influence antibiotic sorption behavior and result in measurable differences in *K_d_* values among sludge types.

In this context, this study aims to systematically investigate the sorption behavior of multiple antibiotics in sludge systems with different physicochemical characteristics. Batch equilibrium experiments were conducted to determine time-dependent *K_d_*, and sludge properties were characterized using BET surface area analysis, SEM, and elemental analysis. The objectives of this study were to (i) evaluate the sorption behavior and equilibrium partitioning of antibiotics, (ii) examine the influence of sludge properties on sorption mechanisms, and (iii) assess the implications of experimentally determined *K_d_* values for predicting antibiotic fate and removal in wastewater treatment systems.

## 2. Materials and Methods

### 2.1. Materials

The stock standard solution including ceftiofur, clopidol, fenbendazole, lincomycin, penicillin G, tiamulin, virginiamycin S1, sulfachloropyridazine, sulfadiazine, sulfadimethoxine, sulfamethazine, sulfamethoxazole, sulfaquinoxaline, sulfathiazole, and trimethoprim was prepared in methanol. Fenbendazole-d3 was used as the internal standard. KH_2_PO_4_, K_2_HPO_4_, Na_2_HPO_4_, NH_4_Cl, MgSO_4_, CaCl_2_, FeCl_3_, NaHCO_3_ were used for making synthetic wastewater. All standard chemicals were purchased from Sigma-Aldrich ((St. Louis, MO, USA)). The methanol and water used in experiments were HPLC grade (J.T. Baker, Phillipsburg, NJ, USA).

The sludges used in this study were collected from two municipal wastewater treatment plants located in the southern and northern regions of the Seoul metropolitan area in Korea. Both wastewater treatment plants primarily receive municipal wastewater originating from residential and commercial areas and operate conventional activated sludge treatment processes. Sludge A was collected from a treatment plant located in the southern metropolitan region, whereas sludge B originated from a treatment plant located in the northern metropolitan region. Differences in wastewater composition and influent characteristics may contribute to variations in sludge physicochemical properties. The samples are hereafter referred to as sludge A (southern region) and sludge B (northern region), respectively. The sludges were collected in polypropylene (PP) containers. Collected sludges were transported to the laboratory and dried in a 103 °C oven for 24 h for microbial inactivation. Sludge samples were thermally treated to suppress microbial activity, ensuring that observed removal was attributable solely to sorption rather than biodegradation. The dried sludges were homogenized using a mortar and pestle and sieved (1.00 mm). The sieved sludges were stored in glass bottles before use.

### 2.2. Sorption Experiments

#### 2.2.1. Sorption Experimental Procedure

Sorption kinetic experiments were conducted with sludge. The initial aqueous solution used in the adsorption experiment was prepared by adding a stock standard solution to synthetic wastewater to a concentration of 10 µg/L. Based on previous studies, sorption equilibrium for many micropollutants in sludge systems can be achieved within relatively short time periods [17]. In addition, preliminary evaluation of concentration changes indicated that most compounds approached stable concentrations after approximately 24 h. Therefore, a 24 h incubation time was considered sufficient to represent operational equilibrium conditions in this study. For the next step, 0.15 g (dry weight) of thermally inactivated sludge and 50 mL of the initial aqueous solution were put in the 50 mL polypropylene tube. The tubes were shaken in 23 °C incubator under 60 rpm. Each tube was equilibrated during 0.4, 2.5, 4, 24 h and all experiments were conducted in quadruplicate (*n* = 4) for each time point. The samples were centrifuged at 2800 *g* for 5 min and the supernatants were separated using a pipette. The solid–water distribution coefficient (*K_d_*, L/kg) was calculated using the following equation:

$$K_{d}=\;\frac{C_{0}-C_{w}}{C_{w}}\frac{V}{M}$$

where C_0_ is the measured aqueous concentration in the corresponding blank sample (µg/L), C_w_ is the aqueous concentration after equilibration with sludge (µg/L), V is the solution volume (L), and M is the dry mass of sludge (kg). For *K_d_* calculations, C_0_ was defined as the measured concentration in the corresponding blank sample collected at the same sampling time rather than the nominal spike concentration (10 μg/L). This approach accounts for concentration variations occurring in the absence of sludge and provides a more accurate estimate of sorption behavior.

#### 2.2.2. Sample Preparation

The supernatants were treated using Oasis HLB SPE cartridge (200 mg, 6 mL; Waters, Milford, MA, USA). The cartridge was preconditioned with 5 mL of Milli-Q water. A sample was then loaded into a cartridge with the internal standard solution. The cartridge was dried by using a vacuum pump for 30 min and eluted with 10 mL of methanol. The extracts were transferred to 2 mL vials.

#### 2.2.3. Instrument Analysis

Target analysis of 15 antibiotics was conducted using HPLC-MS/MS 8040 (Shimadzu, Japan). 3 μL of sample was injected and analyzed using an analytical column (Eclicpse Plus C18 (I.D. 3.0 mm × L. 150 mm, 3.5 μm); Agilent, Santa Clara, CA, USA). The flow rate in the mobile phase was 0.2 mL/min. The nebulizing gas flow rate was maintained at 2 L/min and the drying gas flow rate at 15 L/min. For a quantitative analysis of the target ions, the multiple reaction monitoring (MRM) analysis positive ion electrospray (ESI) mode was used. Analyses were performed in positive ESI mode with a DL temperature of 250 °C. For each target compound, one MRM transition was used for quantification and a second MRM transition was used for confirmation. The precursor ion, quantifier ion, and qualifier ion used for LC-MS/MS analysis are provided in Table S2. The mobile phase consists of (A) 0.1% formic acid in water and (B) 0.1% formic acid in acetonitrile. The binary gradient was set based on (B): maintain 5% (0–0.5 min), increase from 5% to 60% (0.5–5.5 min), increase from 60% to 100% (5.5–13.0 min), maintain 100% (13–16 min), decrease from 100% to 5% (16.0–16.2 min), maintain 5% (16.2–17 min). Antibiotics were quantified in the aqueous supernatant following sorption equilibration, and no direct extraction of antibiotics from sludge solids was performed in this study.

### 2.3. Quality Assurance and Quality Control

The pure methanol as blank samples were analyzed after every 10 samples to check for instrument background contaminations. Blank experiments without sludge were conducted under identical conditions to account for potential losses due to adsorption to container walls or degradation. Sorption was evaluated by comparing aqueous concentrations between sludge-treated samples and corresponding blanks. Because all samples, blanks, and calibration standards were processed using identical sample preparation and analytical procedures, quantitative comparisons were performed under consistent analytical conditions. Calibration curves were prepared using five concentration levels (0.01, 0.025, 0.05, 0.08, and 0.10 mg/L). The target compounds exhibited good linearity, with coefficients of determination (R^2^) ranging from 0.9604 to 0.9997. Because matrix-matched calibration and matrix-effect assessments were not performed, the potential influence of matrix-related analytical effects should be considered when interpreting the quantitative results.

## 3. Results and Discussion

### 3.1. Time-Dependent Sorption Behavior and Distribution Coefficients of Antibiotics

**Figure 1:**
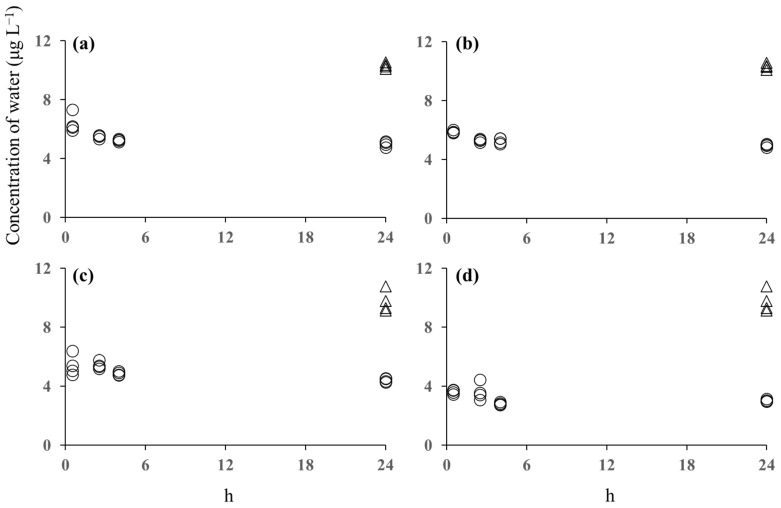
Time-dependent changes in aqueous concentrations (μg L^−1^) of (**a**,**b**) fenbendazole and (**c**,**d**) tiamulin. Panels (**a**) and (**c**) correspond to sludge A, while panels (**b**) and (**d**) correspond to sludge B. Circles (○) represent sludge-treated samples, and triangles (△) represent blank samples without sludge. Data points indicate individual replicates (*n* = 4).

The time-dependent sorption behavior of antibiotics in sludge systems was evaluated using raw aqueous concentration data (Table S1). Overall, a rapid decrease in aqueous concentrations was observed within the initial hours, followed by a gradual approach to equilibrium. This pattern is consistent with the commonly reported two-stage sorption behavior, consisting of a rapid surface adsorption phase followed by a slower intraparticle diffusion process [18]. Representative examples of fenbendazole and tiamulin are shown in Figure 1. In all cases, aqueous concentrations in sludge-treated samples (○) decreased substantially compared to the corresponding blank samples (△), confirming that the observed loss was primarily due to sorption rather than experimental artifacts. Notably, the decrease was most pronounced within the first 4 h, after which concentrations remained relatively stable up to 24 h, supporting the assumption that equilibrium was reached within the experimental timeframe. A clear difference in sorption behavior between the two sludge types was observed. For both compounds, lower aqueous concentrations were consistently measured in sludge B compared to sludge A at equilibrium, indicating a higher sorption capacity of sludge B. This trend was consistently observed across most antibiotics tested (Table S1), indicating that sludge-specific properties were an important factor controlling sorption capacity, although compound-specific properties also contributed to the observed variability.

Based on the equilibrium concentrations at 24 h, *K_d_* were calculated for all antibiotics (Table 1). The *K_d_* values ranged widely depending on the compound, reflecting differences in physicochemical properties such as polarity, ionization state, and functional groups. In general, higher *K_d_* values were obtained for sludge B than for sludge A, indicating stronger sorption affinity in the former. For example, tiamulin exhibited a *K_d_* of 402 ± 53 L/kg in sludge A, compared to 737 ± 76 L/kg in sludge B, while similar trends were observed for most sulfonamides and macrolide compounds. Despite this overall trend, some compounds (e.g., clopidol) showed relatively high variability, as indicated by large standard deviations. This variability may reflect analytical uncertainty and compound-specific sensitivity during concentration measurements, particularly for compounds showing relatively unstable responses at low aqueous concentrations. The results demonstrate that sorption of antibiotics onto sludge is rapid and reaches equilibrium within 24 h, and that sludge B exhibits consistently higher sorption capacity than sludge A. These findings suggest that sludge-specific properties play a critical role in governing the environmental partitioning of antibiotics.

### 3.2. Influence of Sludge Physicochemical Properties on Sorption Behavior

#### 3.2.1. Surface Area and Pore Structure Characteristics (BET Analysis)

The physicochemical properties of the sludge samples were characterized using BET analysis, and the results are summarized in Table 2. Sludge A exhibited a higher specific surface area (0.312 m^2^/g) and total pore volume (0.00292 cm^3^/g) compared to sludge B (0.213 m^2^/g and 0.00166 cm^3^/g, respectively). The relatively low BET surface areas observed in this study may be attributed to the specific drying conditions and the complex composition of sludge samples. In general, a higher surface area and pore volume are expected to enhance sorption capacity by providing more available adsorption sites [19]. However, the sorption results (Table 1) showed an opposite trend, with sludge B consistently exhibiting higher *K_d_* for most antibiotics compared to sludge A. This discrepancy indicates that sorption in this study was not governed solely by physical surface area, but rather by additional factors related to pore structure and chemical interactions.

One possible explanation is the difference in pore structure between the two sludges. Microporous domains can enhance sorption through confinement effects, where organic molecules are retained within small pore spaces, limiting their mobility and reducing desorption potential. This behavior is consistent with previous studies demonstrating that soil organic matter contains internal porous domains where diffusion and sorption processes are strongly coupled [20]. In addition to pore size distribution, structural changes in organic matter may also contribute to the observed behavior. Previous studies have shown that the physical state of organic matter can vary depending on environmental conditions, where drying or structural compaction can lead to shrinkage of organic domains and increased exposure of hydrophobic regions [21,22,23]. Such shrinkage can enhance sorption affinity by creating condensed sorption domains within the organic matrix. Furthermore, the organic fraction of sludge, which shares many characteristics with soil organic matter, acts as the primary sorption phase for organic contaminants. The sorption capacity of sludge is therefore strongly influenced by the internal structure and physicochemical state of the organic matrix [20]. Structural densification and reduction in pore size within the organic matrix can promote pore-filling mechanisms and physical entrapment of compounds, thereby enhancing retention.

In this study, sludge B exhibited a smaller average pore size (31–38 nm) compared to sludge A (37–43 nm), suggesting a denser and more compact pore structure. Such structural characteristics may limit diffusion into deeper domains but enhance the retention of antibiotics once sorbed. This interpretation is consistent with the kinetic results (Figure 1), where rapid initial sorption was followed by stable equilibrium concentrations, particularly in sludge B. These findings indicate that sorption of antibiotics onto sludge is influenced not only by the quantity of available surface area, but also by the quality of adsorption domains and the structural characteristics of the pore network. Therefore, a higher surface area does not necessarily result in higher sorption capacity, particularly for polar and ionizable compounds such as antibiotics.

#### 3.2.2. Elemental Composition and Its Role in Sorption (EDS Analysis)

The elemental composition of the sludge samples, determined by SEM–EDS, is presented in Table 3. Sludge A was characterized by higher contents of carbon (48.19 ± 0.94 wt%) and nitrogen (8.99 ± 0.59 wt%), indicating a relatively higher organic matter fraction. In contrast, sludge B exhibited significantly higher concentrations of inorganic and metal-associated elements, including Fe (1.74 ± 0.32 wt%), Ca (2.37 ± 0.57 wt%), Si (2.28 ± 0.73 wt%), and S (2.26 ± 0.69 wt%). These results suggest that sludge A is predominantly organic, whereas sludge B contains a greater proportion of mineral and metal-rich components. The higher sorption capacity observed in sludge B (Table 1) may be associated with these compositional differences. Metal elements such as Fe, Al, and Ca are known to enhance sorption through mechanisms including complexation, electrostatic interactions, and cation bridging with functional groups present in antibiotic molecules. In particular, the elevated Fe and Ca contents in sludge B may contribute to stronger binding interactions and enhanced sorption affinity. However, the present study does not directly demonstrate the specific mechanisms responsible for the observed differences.

In contrast, although sludge A exhibited a higher specific surface area (Table 2), its relatively lower metal content suggests that sorption was dominated by weaker interactions such as hydrophobic partitioning or surface adsorption onto organic matter. This explains why a higher surface area did not correspond to higher sorption capacity in this study. These findings suggest that the sorption of antibiotics onto sludge is influenced by chemical composition in addition to surface area. Differences in metal-associated components may contribute to the observed higher sorption behavior in sludge B; however, additional studies are required to confirm the specific mechanisms involved.

#### 3.2.3. Morphological Characteristics of Sludge (SEM Analysis)

**Figure 2:**
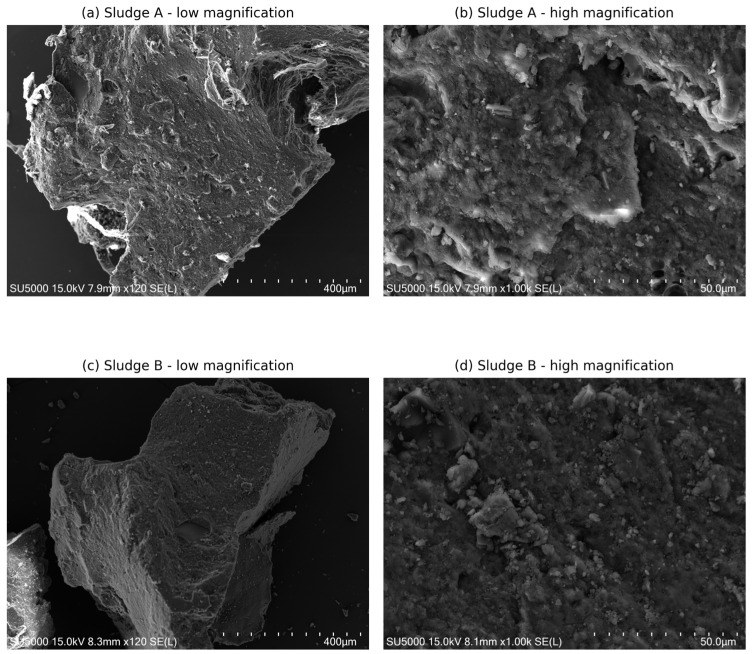
SEM images showing the morphology of sludge A and sludge B at low and high magnifications. Panels (**a**) and (**c**) show low-magnification images, whereas panels (**b**) and (**d**) show high-magnification images. Scale bars are provided within each image.

The morphological characteristics of sludge samples were examined using scanning electron microscopy (SEM), and representative images are presented in Figure 2. Clear differences in structure were observed between sludge A and sludge B. Sludge A exhibited a relatively loose and porous floc structure at low magnification (Figure 2a), with irregularly shaped aggregates and open spaces between particles. At higher magnification (Figure 2b), the surface appeared rough and heterogeneous, with a network-like structure and visible voids.

In contrast, sludge B also exhibited an aggregated morphology (Figure 2c). Differences in particle arrangement and surface texture were observed compared to sludge A. At higher magnification (Figure 2d), sludge B showed numerous fine particulate features distributed across the surface. Although morphological differences between the two sludge samples were visually apparent, the SEM images provide only qualitative information and do not allow definitive conclusions regarding structural compactness or pore characteristics. Overall, both sludge samples exhibited heterogeneous surface morphologies with distinct structural features.

### 3.3. Comparison of Sorption Coefficients with Previous Studies

The *K_d_* values obtained in this study were compared with previously reported values to evaluate the sorption behavior of antibiotics in sludge systems. The measured *K_d_* values (74–737 L/kg) fall within the wide range reported in the literature, which spans from less than 1 to several thousand L/kg depending on compound class and environmental matrix [2,11]. For sulfonamide antibiotics, relatively low sorption affinity has generally been reported due to their hydrophilic nature and ionizable functional groups [8]. For example, Yang et al. (2011) reported *K_d_* values of 28.6–110 L/kg for sulfonamides in activated sludge systems [15], while even lower values (approximately 5–10 L/kg) have been observed in soil environments [24]. Similar trends have also been observed in environmental monitoring studies, where sulfonamides are frequently detected in aqueous phases due to their low sorption affinity [1,25].

In comparison, the *K_d_* values obtained in this study for sulfonamides (74–257 L/kg) are noticeably higher than those reported in previous studies, indicating enhanced sorption capacity of the investigated sludge samples. Such differences can be attributed to the complex composition of sludge matrices. Compared to soils, sludge-derived materials generally exhibit enhanced sorption capacity due to higher organic matter content and the presence of reactive functional groups [13]. In addition, sorption of antibiotics is not governed solely by hydrophobic partitioning but also involves electrostatic interactions, cation exchange, and surface complexation processes [11]. The presence of metal-associated components further enhances sorption through cation bridging and complex formation, as demonstrated for tetracycline interactions with metal oxides [14].

The *K_d_* values obtained in the present study were generally within the range reported for antibiotics in activated sludge and other organic-rich environmental matrices. Previous studies have demonstrated that antibiotic partitioning behavior is highly dependent on both compound-specific properties and sludge characteristics, including organic matter content, pore structure, and mineral-associated sorption sites [11,15,26]. Variations in reported *K_d_* values among studies may also reflect differences in sludge origin, treatment processes, experimental conditions, and analytical approaches [4,5,26]. Recent reviews have further emphasized that sorption remains one of the major mechanisms governing antibiotic removal and environmental fate in wastewater treatment systems, although the relative contribution of sorption varies considerably among antibiotic classes [5,26]. The results obtained in this study support these observations and demonstrate that differences in sludge physicochemical properties can contribute to substantial variability in antibiotic partitioning behavior.

### 3.4. Implications for Modeling and Environmental Fate

In biological wastewater treatment systems, the removal of trace organic compounds is governed by a combination of sorption onto sludge and biotransformation processes [17]. In this study, inactivated sludge was used to isolate sorption, enabling direct evaluation of *K_d_* independent of biological transformation. The experimentally determined *K_d_* values can be directly applied to estimate the fraction of antibiotics removed via sludge partitioning using a solid–water equilibrium model [12,27]. The partition coefficient describes the equilibrium distribution between the aqueous and solid phases, and the fraction of compounds associated with sludge can be expressed as:

$$f_{s}=\frac{K_{d}X}{1+K_{d}X}$$

where fs is the fraction sorbed to sludge, *K_d_* is the distribution coefficient (L/kg), and X is the mixed liquor suspended solids (MLSS) concentration (kg/L). This approach has been widely used to estimate sorption behavior in activated sludge systems, where typical MLSS concentrations range from 2 to 4 g/L [28,29]. Based on this relationship, the *K_d_* values obtained in this study (74–737 L/kg) correspond to a wide range of sorption-driven removal efficiencies. For example, assuming an MLSS concentration of 3 g/L, compounds with moderate sorption affinity (*K_d_* ≈ 100 L/kg) are expected to exhibit approximately 20–30% removal via sludge partitioning, whereas strongly sorbing compounds (*K_d_* ≈ 700 L/kg) may achieve removal efficiencies of up to ~70%. Similar magnitudes of sorption contribution have been reported in previous studies, highlighting the importance of partitioning processes in wastewater treatment systems. These results demonstrate that sorption alone can account for a substantial fraction of antibiotic removal, particularly for compounds with high affinity for sludge. Conversely, more hydrophilic compounds with low *K_d_* values are likely to remain in the aqueous phase and be removed primarily through biotransformation or discharged in effluents [15,29].

Furthermore, sludge characteristics play a critical role in determining sorption behavior. Variations in sludge composition, including organic matter content and metal-associated components, can significantly influence *K_d_* values and thus affect predicted removal efficiencies [13,14]. This suggests that the use of constant partition coefficients in predictive models may oversimplify real systems, and that incorporating sludge-specific parameters could improve model accuracy [16]. Overall, this study demonstrates that sorption is a key mechanism controlling the environmental fate of antibiotics in wastewater treatment systems. The integration of experimentally determined *K_d_* values with simple equilibrium-based models provides a practical framework for estimating removal efficiency and predicting contaminant distribution under realistic operating conditions.

## 4. Conclusions

This study evaluated the sorption behavior of 15 antibiotics in two sewage sludge samples with contrasting physicochemical characteristics and determined their equilibrium distribution coefficients (*K_d_*). The measured *K_d_* values ranged from 74 to 737 L/kg, and for most compounds, higher sorption affinities were observed in sludge B than in sludge A. Despite exhibiting a lower BET surface area, sludge B consistently showed greater sorption capacity, suggesting that factors beyond surface area may influence antibiotic partitioning behavior.

Characterization of the sludge samples indicated that differences in pore structure and elemental composition may contribute to the observed variability in sorption behavior. In particular, sludge B contained higher proportions of metal-associated components and exhibited a more compact pore structure. However, the specific mechanisms responsible for the enhanced sorption could not be directly confirmed in the present study and require further investigation.

Application of an equilibrium-based model demonstrated that sorption can contribute substantially to antibiotic removal in wastewater treatment systems and that sludge-specific properties may significantly influence predicted partitioning behavior. These findings highlight the importance of considering both sludge characteristics and compound-specific properties when assessing the environmental fate of antibiotics.

Several limitations should be acknowledged. The reported *K_d_* values were determined using a single initial concentration and thermally treated sludge, and therefore should be interpreted as equilibrium partitioning parameters under the experimental conditions employed. Because thermal drying may alter sludge physicochemical properties, direct extrapolation of the results to native wet sludge should be made with caution. Future studies employing minimally altered sludge matrices, multiple concentration levels, and additional characterization of sludge properties are needed to improve mechanistic understanding of antibiotic sorption processes in wastewater treatment systems.

## Figures and Tables

**Table 1:** Distribution coefficients (*K_d_*, L/kg dry weight) of antibiotics in sludge A and sludge B. Values represent mean ± standard deviation (*n* = 4).

Chemicals	A	B
Ceftiofur	424 ± 125	534 ± 82
Clopidol	165 ± 176	77 ± 157
Fenbendazole	355 ± 25	361 ± 18
Lincomycin	150 ± 19	213 ± 26
Penicillin G	477 ± 260	621 ± 307
Tiamulin	402 ± 53	737 ± 76
Virginiamycin S1	294 ± 58	487 ± 77
Sulfachloropyridazine	141 ± 37	232 ± 47
Sulfadiazine	74 ± 31	131 ± 43
Sulfadimethoxine	188 ± 21	257 ± 33
Sulfamethazine	194 ± 31	246 ± 45
Sulfamethoxazole	175 ± 20	248 ± 40
Sulfaquinoxaline	162 ± 32	250 ± 38
Sulfathiazole	266 ± 39	314 ± 46
Trimethoprim	315 ± 44	332 ± 33

**Table 2:** BET surface area and pore characteristics of sludge A and sludge B.

Parameter	Sludge A	Sludge B
BET surface area (m^2^/g)	0.312	0.213
Total pore volume (cm^3^/g)	0.00292	0.00166
Micropore area (m^2^/g)	0.105	0.116
External surface area (m^2^/g)	0.208	0.098
Average pore size (nm)	37–43	31–38

**Table 3:** Elemental composition of sludge A and sludge B determined by SEM–EDS (wt%).

Element	Sludge A (wt%)	Sludge B (wt%)
C	48.19 ± 0.94	43.42 ± 1.00
O	29.56 ± 1.70	28.29 ± 2.90
N	8.99 ± 0.59	7.40 ± 0.71
P	2.42 ± 0.25	2.26 ± 0.70
Al	2.01 ± 0.18	2.64 ± 0.49
Si	0.77 ± 0.10	2.28 ± 0.73
Ca	0.89 ± 0.17	2.37 ± 0.57
S	0.66 ± 0.09	2.26 ± 0.69
Fe	0.22 ± 0.13	1.74 ± 0.32
Mg	0.32 ± 0.04	0.58 ± 0.39
Na	0.10 ± 0.01	0.62 ± 0.12
K	0.68 ± 0.12	0.50 ± 0.14
Zn	–	0.58 ± 0.11
Cl	–	0.20 ± 0.05

## Data Availability

The original contributions presented in this study are included in the article/Supplementary Material. Further inquiries can be directed to the corresponding authors.

## Supplementary Materials

The following supporting information can be downloaded at: https://www.mdpi.com/article/10.3390/jox16030112/s1. Table S1: Raw aqueous concentration (μg L^−1^) data for blank and sludge-treated samples over time; Table S2: MRM transitions used for LC-MS/MS analysis of target antibiotics and internal standard. For each compound, one transition was used for quantification and a second transition was used for confirmation; File S1: The original images of Figure 2.
