# Supplementary material for: Sorption of Antibiotics in Sewage Sludge: Distribution Coefficients, Sludge Characteristics, and Implications for Environmental Fate
Source: J Xenobiot. 2026 Jun 14;16(3):112. doi: 10.3390/jox16030112 (PMC13300974; doi:10.3390/jox16030112)
Supplement: Supplementary file 1 [file jox-16-00112-s001.zip › jox-4346073-supplementary-proof.pdf]

Supplementary Materials: Sorption of Antibiotics in Sewage Sludge: Distribution Coefficients, Sludge Characteristics, and Implications for Environmental Fate

Wonsik Shin, Pil-Gon Kim and Min-Ho Oak

Table S1. Raw aqueous concentration (μg L^{-1}) data for blank and sludge-treated samples over time.

	Time(h)	Ceftiofur	Clopidol	Fenbendazole	Lincomycin	Penicillin G	Tiamulin	Virginiamycin s1	Sulfachloropyridazine	Sulfadiazine	Sulfadimethoxine	Sulfamethazine	Sulfamethoxazole	Sulfaquinoxaline	Sulfathiazole	Trimethoprim
**Blank**	24	9.08	13.61	10.29	9.51	5.94	9.14	6.93	9.78	7.45	11.70	10.31	9.73	10.17	9.01	6.66
	24	9.48	16.69	10.39	10.23	10.66	10.78	8.82	11.68	7.95	12.42	10.75	10.31	11.91	10.33	7.07
	24	8.22	7.93	10.09	9.96	6.08	9.31	7.03	10.86	7.33	11.30	9.92	10.40	10.47	9.39	6.80
	24	9.21	7.65	10.56	9.68	5.24	9.80	8.41	10.65	8.58	11.65	10.98	10.62	10.59	10.02	7.41
**A**	0.5	5.79	7.30	6.20	6.30	2.81	5.07	5.89	8.88	6.04	8.60	7.47	7.56	8.71	5.66	3.20
	0.5	5.84	7.98	7.34	7.24	2.99	5.42	6.13	9.48	6.52	8.86	8.85	8.30	9.52	6.64	3.88
	0.5	5.01	5.97	5.92	6.42	2.84	4.78	5.91	8.11	7.04	7.63	7.55	7.11	8.21	5.65	3.48
	0.5	7.09	8.09	6.14	7.88	3.64	6.41	6.91	9.44	7.95	9.19	9.09	8.70	9.20	7.06	4.32
	2.5	7.12	15.12	5.57	8.07	3.56	5.22	6.14	10.44	7.74	8.80	8.09	8.97	9.09	6.90	4.10
	2.5	6.66	8.25	5.50	7.28	3.17	5.41	5.63	10.42	6.79	9.03	8.12	7.89	8.98	6.76	3.68
	2.5	6.08	7.77	5.33	7.38	2.99	5.34	5.23	9.64	6.98	8.48	7.88	7.65	8.58	6.27	3.63
	2.5	5.53	7.20	5.58	8.21	3.21	5.79	5.25	10.00	7.39	8.76	7.80	8.44	9.00	6.35	4.14
	4	3.91	8.68	5.27	7.83	3.65	4.95	5.12	8.45	6.96	8.23	7.20	8.11	8.26	6.46	3.91
	4	3.46	10.76	5.24	7.63	3.56	4.78	4.83	8.22	7.40	8.10	7.66	7.89	7.97	6.40	3.69
	4	3.70	7.23	5.36	7.01	3.41	5.04	4.86	8.33	7.35	8.07	7.30	7.84	7.95	6.10	3.81
	4	4.28	7.33	5.16	7.15	3.44	4.76	4.78	8.08	7.16	8.37	7.49	7.20	8.07	6.03	3.73
	24	4.44	7.40	4.76	6.55	2.85	4.28	4.07	7.50	6.03	7.29	6.56	6.65	7.15	5.24	3.51
	24	3.30	8.00	5.16	7.01	2.84	4.50	4.06	7.34	6.53	7.58	7.03	6.70	7.18	5.63	3.83
	24	4.83	8.32	5.12	6.91	3.09	4.55	4.14	8.13	6.65	7.65	6.70	6.63	7.39	5.50	3.71
	24	3.65	7.09	4.98	6.73	2.73	4.36	3.98	7.28	6.46	7.60	6.28	6.96	7.34	5.20	3.35

**B**	**0.5**	**4.24**	**6.55**	**5.87**	**6.50**	**3.45**	**3.60**	**4.05**	**6.84**	**6.37**	**7.35**	**6.74**	**6.01**	**7.29**	**6.25**	**4.27**
	**0.5**	**4.04**	**6.80**	**5.85**	**6.42**	**3.32**	**3.78**	**4.17**	**6.67**	**6.12**	**6.74**	**5.76**	**6.80**	**7.05**	**5.89**	**4.28**
	**0.5**	**3.96**	**7.68**	**5.82**	**6.27**	**3.42**	**3.74**	**4.21**	**7.05**	**6.64**	**7.26**	**6.51**	**6.37**	**6.57**	**6.11**	**4.16**
	**0.5**	**4.06**	**7.15**	**6.04**	**5.84**	**3.16**	**3.48**	**3.94**	**6.70**	**5.63**	**7.21**	**6.48**	**6.34**	**6.85**	**5.63**	**3.99**
	**2.5**	**4.28**	**8.06**	**5.41**	**6.55**	**3.05**	**3.44**	**4.06**	**7.05**	**6.11**	**7.35**	**6.29**	**7.07**	**7.00**	**5.77**	**4.06**
	**2.5**	**5.10**	**9.05**	**5.29**	**6.60**	**3.36**	**4.45**	**4.09**	**7.48**	**7.41**	**7.51**	**6.93**	**7.19**	**7.13**	**5.71**	**4.16**
	**2.5**	**6.22**	**11.07**	**5.33**	**6.80**	**3.34**	**3.57**	**4.02**	**7.44**	**6.77**	**7.88**	**7.13**	**7.48**	**7.43**	**6.43**	**4.34**
	**2.5**	**4.07**	**7.73**	**5.16**	**6.16**	**3.00**	**3.09**	**3.68**	**6.84**	**6.21**	**7.02**	**6.09**	**6.28**	**6.55**	**6.33**	**3.83**
	**4**	**2.81**	**9.61**	**5.08**	**5.55**	**3.49**	**2.73**	**3.33**	**5.85**	**6.02**	**6.48**	**5.53**	**6.04**	**5.98**	**5.81**	**3.60**
	**4**	**3.47**	**9.59**	**5.18**	**5.85**	**3.79**	**2.85**	**3.50**	**6.87**	**5.51**	**6.69**	**5.64**	**6.40**	**6.48**	**5.42**	**3.76**
	**4**	**3.31**	**8.89**	**5.44**	**5.80**	**3.54**	**2.78**	**3.52**	**6.87**	**5.89**	**6.98**	**5.77**	**5.96**	**6.57**	**6.09**	**3.63**
	**4**	**3.68**	**x**	**5.44**	**6.08**	**3.73**	**2.95**	**3.68**	**6.83**	**6.67**	**6.98**	**6.74**	**7.00**	**6.78**	**5.62**	**4.02**
	**24**	**3.72**	**7.75**	**4.97**	**5.74**	**2.65**	**3.06**	**3.01**	**6.23**	**5.34**	**6.51**	**5.78**	**5.71**	**6.11**	**4.92**	**3.41**
	**24**	**3.23**	**8.72**	**4.82**	**5.90**	**2.34**	**3.14**	**3.08**	**5.93**	**5.42**	**6.42**	**5.70**	**5.52**	**6.20**	**4.77**	**3.41**
	**24**	**x**	**11.74**	**5.00**	**6.06**	**2.34**	**2.97**	**3.16**	**6.84**	**6.31**	**7.15**	**6.74**	**6.48**	**6.20**	**4.94**	**3.59**
	**24**	**x**	**9.93**	**5.07**	**6.36**	**2.45**	**2.99**	**3.20**	**6.39**	**5.50**	**6.57**	**6.04**	**5.91**	**x**	**5.38**	**3.59**

x = unavailable analytical data due to instrumental or analytical issues; values were excluded from statistical calculations.

Table S2. MRM transitions used for LC-MS/MS analysis of target antibiotics and internal standard. For each compound, one transition was used for quantification and a second transition was used for confirmation.

Analytis	Precursor Ion	Quantifier Ion	Qualitative Ion
Ceftiofur	523.90	126.20	166.10
Clopidol	191.95	101.15	87.10
Fenbendazole	299.95	268.05	159.10
Lincomycin	407.05	126.30	359.20
Ampicillin	382.15	223.15	333.10
Penicillin-G	325.20	86.30	91.25
Tiamulin	494.35	192.25	119.10
Flumequine	262.05	244.15	202.15
Marbofloxacin	363.10	72.20	320.10
Virginiamycin-S1	824.20	205.10	290.10
Sulfachloropyridazine	285.20	156.10	65.15
Sulfadiazine	251.05	156.15	92.20
Sulfadimethoxine	311.10	156.15	108.15
Sulfamethazine	279.00	186.20	124.15
Sulfamethoxazole	253.90	92.15	156.10
Sulfaquinoxaline	301.10	156.10	92.10
Sulfathiazole	256.00	156.00	92.15
Trimethoprim	291.15	230.15	123.20
Chlortetracycline	479.00	462.10	98.00
Oxytetracycline	461.00	443.00	426.15
Doxycycline	445.00	428.20	154.20
Fenbendazole-d3	302.90	268.00	159.10
