# Supplementary figures and images for: Sorption of Antibiotics in Sewage Sludge: Distribution Coefficients, Sludge Characteristics, and Implications for Environmental Fate
Source: J Xenobiot. 2026 Jun 14;16(3):112. doi: 10.3390/jox16030112 (PMC13300974; doi:10.3390/jox16030112)

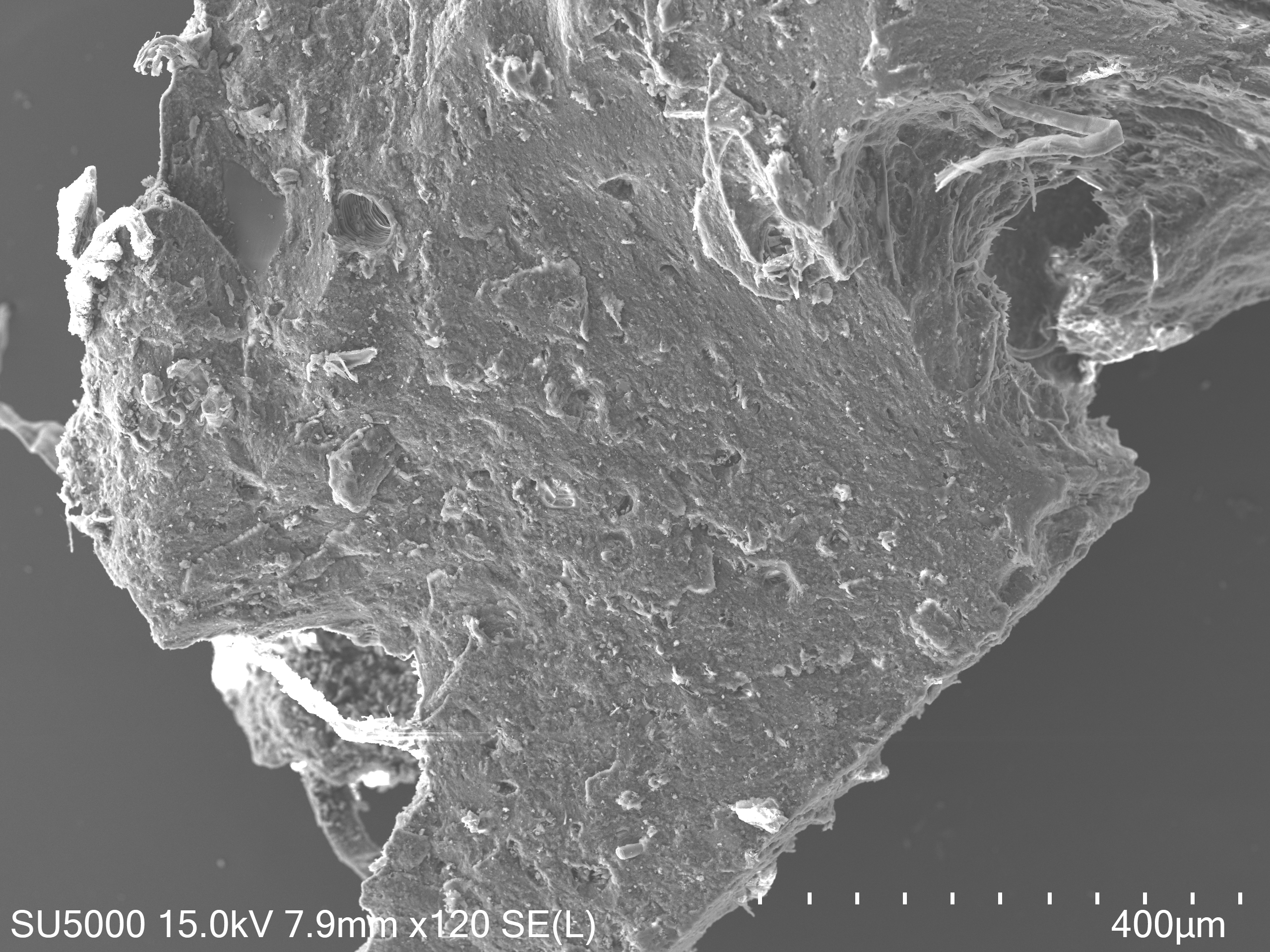

Supplement: Supplementary file 1 [file jox-16-00112-s001.zip › jox-4346073-FileS1-The original images of Figure 2/figure 2a. sludge A low magnification.bmp]

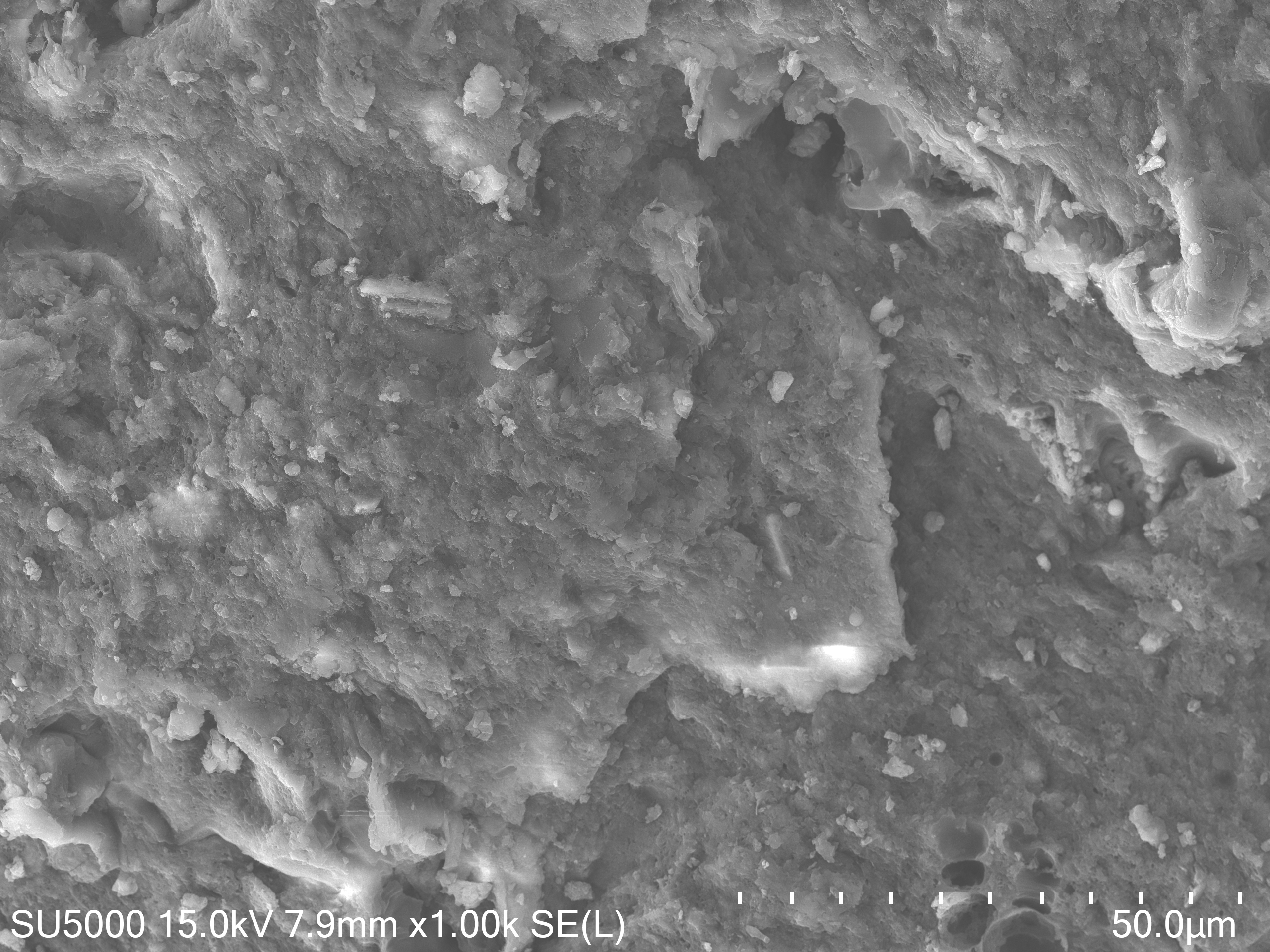

Supplement: Supplementary file 1 [file jox-16-00112-s001.zip › jox-4346073-FileS1-The original images of Figure 2/figure 2b..bmp]

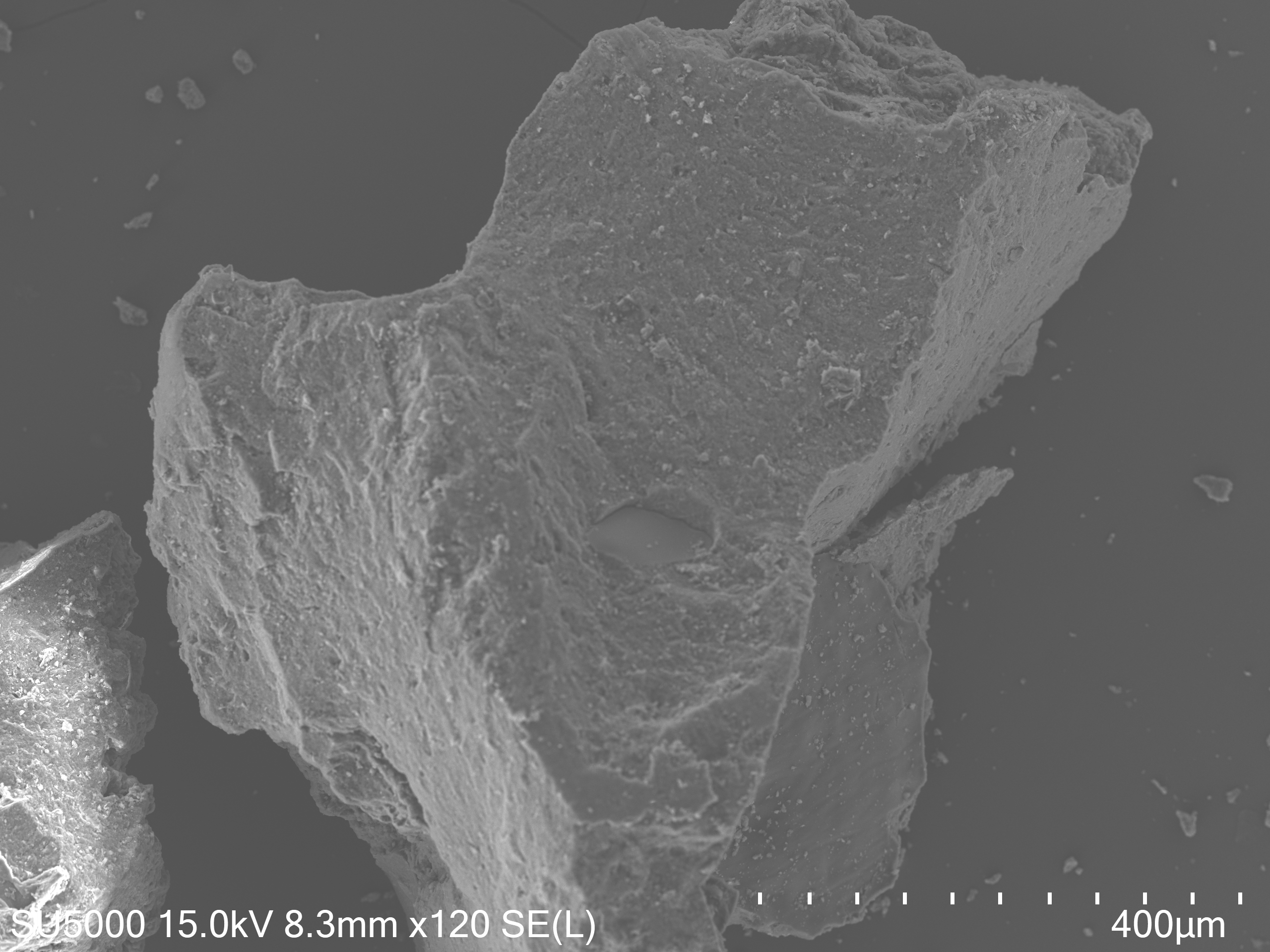

Supplement: Supplementary file 1 [file jox-16-00112-s001.zip › jox-4346073-FileS1-The original images of Figure 2/figure 2c sludge B low magnification.bmp]

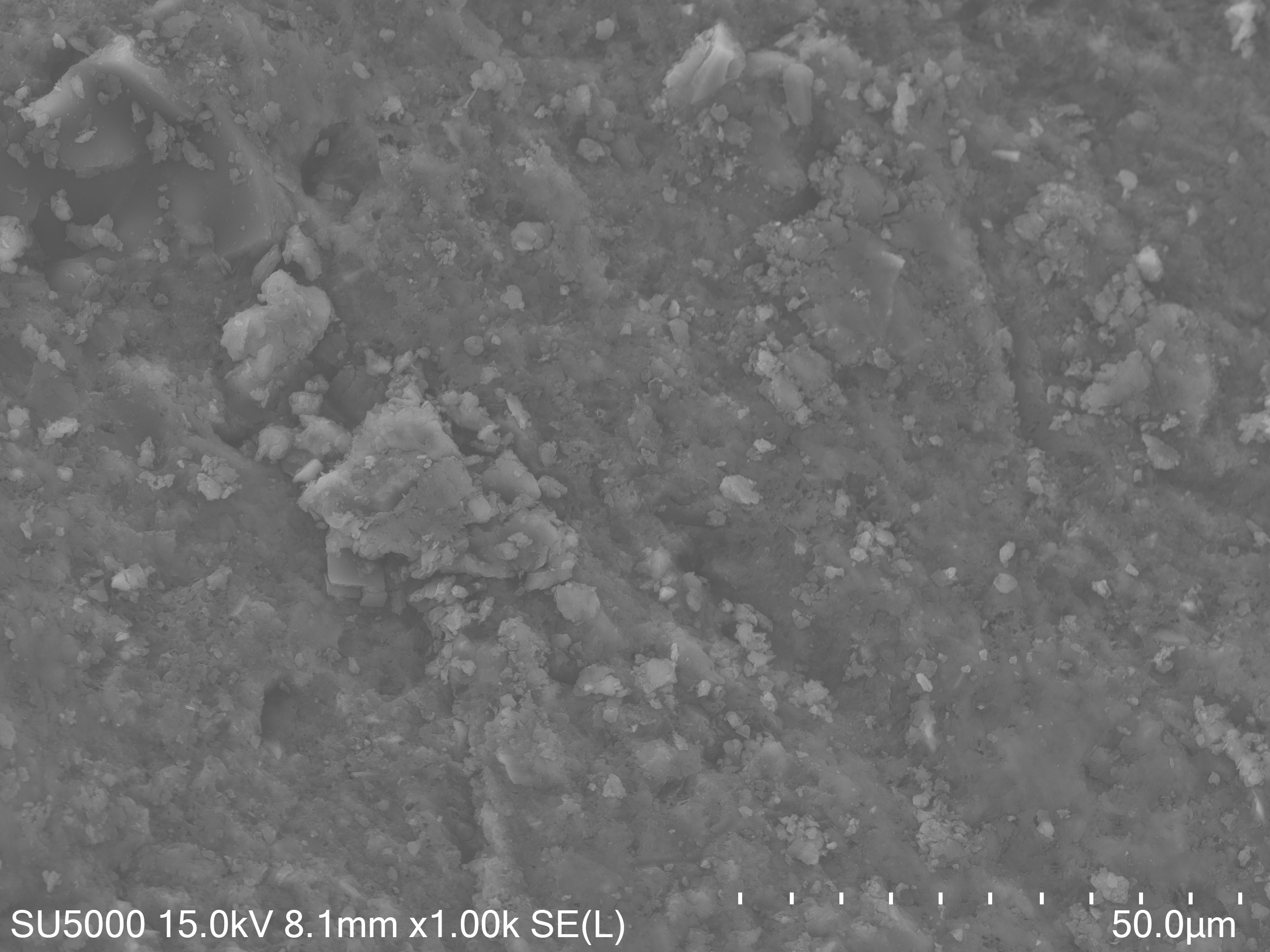

Supplement: Supplementary file 1 [file jox-16-00112-s001.zip › jox-4346073-FileS1-The original images of Figure 2/figure 2d.bmp]
